# Banning Asbestos in New Zealand, 1936–2016, an 80-Year Long Saga

**DOI:** 10.3390/ijerph14121457

**Published:** 2017-11-25

**Authors:** William Ivan Glass, Rob Armstrong, Grace Chen

**Affiliations:** 1 Centre for Public Health Research, Massey University, Wellington 6021, New Zealand; g.chen1@massey.ac.nz; 2 Respiratory Physician, Hawke’s Bay, Hastings 4120, New Zealand; strongarm17a@gmail.com

**Keywords:** asbestos, banning, New Zealand

## Abstract

The banning by the New Zealand Government of the import and export of asbestos-containing products resulted from the interplay of a number of factors. At a personal level, there were the actions of the asbestos sufferers, their families and support groups. At the political level, there were the activities of progressive trade union groups representing the hazardous trades, such as labourers, construction workers and demolition workers, and at a Government level, there was a positive response to these public health pressures. The Prohibition Order 2016 concerning Imports and Exports (asbestos-containing products) was the outcome of this 80-year long saga.

## 1. Introduction

It is unusual for one country to learn from the experience of another country; it is even more unusual for a country to learn from the experience of its’ own past. The decision to ban the importation of asbestos by the New Zealand Government in 2016 [1] took place at a moment in time. The reasons why this decision was made need to be looked for in the interaction of a range of events and influences, which occurred over many decades. 

While such events and influences can be seen in retrospect to have had an incremental effect, together they are not always sufficient. There is a need for something else, a more dramatic often more tragic event or events to bring the legislative process to its conclusion.

It will be suggested that this was the case in New Zealand, when within a short period of six months, the Canterbury region in the South Island suffered two major earthquakes, September 4th, 2010, February 22nd, 2011 and tragically between these two natural disasters, on November 19th, 2010, this time on the West Coast of the South Island, a massive explosion occurred in an underground coal mine. These three events led not only to the loss of many lives but also to huge material destruction, in the case of the earthquakes, buildings, houses, roads and underground reticulation and with the mine, its permanent closure. 

## 2. 1936—The First Awareness of the Health Dangers of Asbestos in New Zealand

Bulletin No. 57 [2] contained the first reference to the dangers of asbestos. Published by the Department of Scientific and Industrial Research, the Chairman of the Committee at the time of publication was Dr.T.R. Ritchie, Director of the Division of Public Hygiene, Department of Health. The reference was as follows.

In the working of asbestos the dust produced gives rise to a pulmonary condition known as asbestosis, which although different in its character from silicosis causes a disease similar in some respects to it.

What is interesting about this comment was that it was only a few years earlier, that Cooke [3,4], Gloyne [5,6], and Merewether [7,8] had highlighted the health dangers of asbestos in a country, the United Kingdom, that had by then been processing and manufacturing asbestos-containing products for some 30 or more years. The New Zealand asbestos era had yet to begin, but that brief paragraph would be seen as remarkably perceptive. Industry and Government had been forewarned. 

## 3. 1936–1966 A Time of Inaction

Although the first asbestos cement plant was established in 1943, there was little further response by Government to the dangers of working with asbestos. With two exceptions, the first, a brief note in a Report to Government on Industrial Hygiene in New Zealand in 1944 [9] as follows:

….exposure to various dusts (organic and mineral, some of the latter, such as silica and asbestos, capable of causing specific chest diseases…).

The Report was based on the author visiting over 200 factories and making specific recommendations; one of which resulted from a visit to the first asbestos cement plant, where he commented on the need for “The provision of local exhaust ventilation in connection with the handling of asbestos [10]”. This comment was subsequently forwarded to the local branch of the Department of Labour for action and a response was recorded a year later as follows “A large hood with an adequate extraction fan has been placed over the mixing plant in which cement and asbestos are used. No further action is necessary [11].”

The second reference to asbestos appeared in 1951, in the Annual Report of the Department of Health [12] under the heading “Asbestos Quarrying”.

Asbestos is now being quarried in the Dominion, and the dust can be expected to cause a certain amount of lung damage unless proper precautions are taken. It produces a somewhat similar condition to silicosis, which is contracted from silica dust, though not nearly as rapidly, nor with such a high incidence of tuberculosis associated with it. On the other hand, asbestos seems to be a more carcinogenic dust than silica.

This chrysotile asbestos was found in commercial quantities of serpentine in the Nelson region. The quarrying continued through to 1963.

Thus, for three decades since that initial warning in 1936, there was little activity by Government alerting industry or the public to the health hazards of asbestos. Even though there were now two major asbestos cement manufacturing plants employing several hundred workers, dairy factories, sawmills and power stations were all using asbestos as an insulation material together with a number of companies spraying asbestos on steel beams and onto the ceilings of homes. Further, with asbestos cement products now readily available, their use was expanding in the construction industry in the domestic, commercial and civic areas. Coinciding with this increasing use of asbestos and compounding the health risk was the introduction of a faster and dustier cutting process in construction—the skill saw.

## 4. 1966–1969 The Asbestos Cement Industry, Christchurch

During this period, the company manufacturing asbestos cement products attracted the attention of both the Department of Health and the Department of Labour. Visits to the Company and communications with the Company and between the two Departments took place on a number of occasions [13,14,15,16], one such was as follows.

At the commencement of the production line, an employee tips bags of asbestos into a hopper and when this is weighed releases it. It would seem to me to be a rather dusty occupation and I wondered if, in view of the possibility of asbestosis, there should not be some form of extraction to protect the employee?

[14]

One wonders what had happened to the report and action in 1944? Had something been forgotten over those 20 years? Or does this illustrate a more general and serious feature of workplace practice? Namely, a lack of an “institutional history”, a failure to record good procedures and experiences, so that each generation has to re-learn safe work methods often at the expense of their health and lives. 

## 5. 1968 The New Zealand Building Worker’s Union

At about this time, the Building Worker’s Union, concerned at the increasing use of asbestos-cement products in the construction industry, published a series of in-depth articles in their union journal, New Zealand Building Worker [17,18,19]. These were based on a visit by the Auckland Branch President to Australia to attend their sister union. It was the first time any organisation, including Government, had alerted the workers, their families and the wider public to the health risks of exposure to asbestos.

Two years later in 1971, the National Secretary of the Union [20] wrote to the Minister of Health challenging the Department’s viewpoint that the health risk to exposed workers in New Zealand was less because of the lower proportion of crocidolite in asbestos materials. The secretary pointed out that this view was now contested.

The building trade unions had taken leadership. Why was this? Was it because working people as the sufferers of a work-related disease are the first to become aware and make the connection between work and disease? Or was it because they exchange experiences and so know of “Jack” or “Tom”, who also developed the disease doing the same job? 

## 6. 1971 A Government Response. The National Asbestos Survey, 1969/1970, Report of the Occupational Health Unit, Department of Health, Wellington

This survey [21] was carried out over the two years 1969/1970 on 201 asbestos workers in New Zealand. The survey included dust counts, chest X-rays and lung function tests. The Report was labelled Restricted, Confidential, Not for publication! 

Prior to the Survey commencing, the Associate Director, Occupational Health, Department of Health, had received a detailed letter from James Hardies’ Chief Medical Officer [22] that had outlined in considerable detail the health and exposure surveillance programme carried out by the Company on their employees, throughout their Australian activities including their Auckland asbestos cement plant.

A brief summary of the Health Department Report showed that using a Threshold Limit Value (TLV) for asbestos of 2 mppcf (million particles per cubic foot), equivalent to 12 fibers per cubic centimetre, 71 workers out of 108 tested were working at unacceptable levels which ranged from 0.3 to 10 mppcf. A total of 101 of the workers were X-rayed; 17 showed pleural changes and there was one case of asbestosis. There was no demonstrable effect of time of exposure on lung function, although there was a measurable effect of smoking and there was a difference between the lung function of Maori (the indigenous Polynesian people of New Zealand) and European workers, a finding which had been demonstrated earlier [23]. It was also recorded that the investigation was difficult to undertake and hard to obtain full co-operation from some companies and some workers. This can be seen in the results.

The outcome was:

1. The industry was encouraged to seek alternatives to asbestos where possible.
2. Employees were to have a comprehensive medical examination and ongoing surveillance.
3. Asbestos regulations were to be developed along the lines of the 1969 Regulations in the United Kingdom.

By the end of this decade, Government, the Building Workers Union and some enlightened companies had begun to focus on asbestos as a work hazard and health risk and to introduce dust control and health surveillance measures.

In 1973, the asbestos cement company at the centre of the initial investigation stopped production of these products. 

## 7. The Asbestos Regulations Proposed 1973, Published 1978

Resulting from the Department of Health survey and recommendations, the Minister of Health [24] wrote to the Minister of Labour recommending the need for specific asbestos regulations and suggesting the 1969 Asbestos Regulations of the United Kingdom be the model. This was agreed [25], with the proviso that the Department of Labour investigates areas of asbestos use outside those included in the 1969/1970 survey, such as stores or warehouses accommodating bulk supplies.

While this process was going through its various stages, there was an increasingly urgent need to respond to union concerns about asbestos in the construction industry. The only legislation available was the Construction Act 1959 and the Construction Regulations 1961, which were not specific to asbestos. The outcome was a decision by the Department of Labour to develop an Asbestos Guide for Safety in the Construction Industry. Meanwhile, work on the regulations continued with the view at this stage being that the cornerstone of the regulations should be a concentration on asbestos levels in air and medical examination of the workers [26]. In 1978, the new regulations were published.

Why was this drafting process so slow? By this time there was sufficient evidence from overseas confirming the health risks of asbestos exposure and this was now well known by the Department of Labour, as this statement indicates [27] “Head Office is fully aware of the dangers connected with spraying, sawing, grinding and processing of this material.”

Government awareness was there, yet there appeared to be no urgency. By developing regulations, the need for further action had diminished, apart from restricting asbestos in air exposure levels from time to time and removing the difference in such levels between amphiboles and chrysotile. 

This one-sided approach based on controlling exposure to asbestos dust at work, when the industry had limited means to measure such exposure or hygiene expertise [28], meant that on the surface it appeared that something practical was being achieved when in fact it was largely on paper. Asbestos continued to be used in the manufacture of asbestos cement products, (until 1986) and to be used in a widespread manner in the construction industry, insulation, brake linings and a range of products.

## 8. 1984–1990 Unions Take the Initiative 

The focus moved from Government to the trade unions, with newspaper reports like the following [29]. 

“Asbestosis risks hidden from quarry workers”. This report referred to the blending of serpentine with limestone for agriculture fertiliser and the failure of the Health Department to inform the workers of the risk. 

Meanwhile, the Engineers Union Journal, Metal [30] ran a full page article “Pinning down a killer—the asbestos story”.

In 1984, a letter was forwarded to the Secretary of the New Zealand Federation of Labour, from the Auckland Trades Council, Trade Union Health and Safety Centre on behalf of two occupational medical specialists expressing concern at the Department of Health procedures for the diagnosis of asbestos-related diseases [31]. 

The Engineers Union continued their activity with a determination to get the film “Alice a Fight for Life” shown on National Television. Again the question must be asked why was it necessary for the Union to take this initiative in the Alice film? Why was not leadership coming from Government?

## 9. 1988–1990 The South Island Labourers’ Union Investigation

This investigation [32] was the Union’s response to an upsurge in public concern about asbestos-related illnesses and deaths concerning their members who had been employed in the local asbestos cement factory. A total of 86 union members were reviewed, all of whom had been employed by this factory at some stage. A total of 13 had died from mesothelioma, 3 from lung cancer, 7 from asbestosis, one from chronic obstructive pulmonary disease (COPD) and one from pancreatic cancer. Of those still living, one had mesothelioma, 3 lung cancer, 4 non-lung cancer, 6 asbestosis, 21 pleural changes, 8 COPD and 9 lung changes not specified. Bringing together these individual cases over a short period of time from the local hospital together with those from the investigation of new cases identified by the Union questionnaire was a way of creating a greater impact than “drip feeding” them into a reporting system. It also increased the chances of the sufferers gaining some belated compensation.

## 10. 1988–1990 The Victim’s and Their Spouses Speak Out

This outpouring of individual grief, anger and concern coming almost 20 years after the Department of Health’s Report (1969/1970), owed much to the commitment of a few newspaper reporters [33,34,35,36]. Public awareness of asbestos was growing as week after week, harrowing stories appeared of painful deaths from mesothelioma and tearful widows blaming uncaring employers and an inactive Government for failing to deal with a known health risk. The peak of this individual victim concern occurred, when a retired engineer from the Government Electricity Corporation, who, when unable to obtain compensation because of legal anomalies, took private action against the Government.

### 10.1. 1989–1993 The Robin McKenzie Case 

Mr McKenzie was an engineer employed in the State electricity organisation from the age of 31 in 1940 until his retirement in 1981. In 1989 he first suffered respiratory symptoms which resulted in the diagnosis of mesothelioma. Under the current Accident Compensation law at the time he was ineligible to claim as his exposure preceded the Act coming into force in 1974. As a consequence, he sought permission from the Court of Appeal to confirm he had a common law right to take a claim in negligence against the Crown in the High Court. This claim for 2 million dollars, began in 1992, Mr McKenzie still being alive. In 1993 an out of court settlement was reached [37,38]. One outcome was that it was now possible for other sufferers to initiate a legal claim for a disease suffered from exposure prior to 1974, provided the claim was filed before April 1973. Asbestos sufferers now had the option of submitting a claim to the Accident Rehabilitation and Compensation Corporation. This case was pivotal in the developing history of asbestos exposure in New Zealand. 

### 10.2. 1988–1990 The Clarrie and Thelma Bell Case, Christchurch 

This case, like the Robin McKenzie case, also attracted considerable media publicity. Thelma Bell was the wife of Clarrie who was dying from mesothelioma after a lifetime working in the asbestos cement industry. Thelma went public about the circumstances of her husband’s exposure to asbestos [39]. This resulted in workmates of Clarrie adding in equally graphic images their stories of what working conditions were like at that time. 

All this activity from the mid-1980’s to the end of the decade affected Government. Mr Bill Birch the Minister of Labour responded to both the patient and public concerns. There was also another factor, his electorate seat included the Huntly Power Station a coal-fired station “riddled” with asbestos in the form of pipe insulation. Mr Birch was also responding as a good local MP. The outcome was the Report.

## 11. 1991 Asbestos: Report of The Asbestos Advisory Committee to The Minister of Labour

The Minister initiated the report in 1990 and received it in 1991 [40]. The speed with which the report was completed was a tribute to the Convenor Keith McLea, and although like all Reports it did not satisfy everyone. It was a major step forward, acknowledging what was now a major epidemic of asbestos-related disease, with 174 cases of mesothelioma and an unknown number of lung cancers and cases of asbestosis. Results were only going to worsen with time.

One important outcome of the Report was the establishment of a National Asbestos Register in two parts, a Disease Register and an Exposure Register, both voluntary, but both in time indicating their value. To manage the Register, the Department of Labour established a Medical Panel.

## 12. 1992 The Asbestos Register

During the decade following the Report, there was a continuing level of interest and activity by Government [41,42], some large industries, Unions, and Asbestos Support Groups. 

The two parts of the Register, exposure and disease, saw an upsurge in reporting, with the opportunity for the first time, to develop some meaningful statistics on a significant occupational disease. Information was now being collected on industry, occupation, exposure, disease type, gender, ethnicity, smoking and lung function. Annual Reports (1992–2014) and special reports [43,44] were published, as were presentations by visiting overseas experts [45,46,47]. Over time, enthusiasm for notification began to diminish so that the numbers declined. In spite of this, both parts of the register continue now 25 years later, with the exposure register containing over 20,000 names and the disease register 1500, There thus exists a useful research base from which to evaluate the impact on the workers of the uncontrolled exposure to asbestos fifty or more years ago. 

## 13. Asbestos Support Groups

The development of asbestos support groups evolved from the patient and family concerns so dramatically recorded in the press in the late 1980’s. Groups were established in Wellington, Christchurch and Auckland [48]. Their functions included help and advice to families in terms of how to proceed with medical help and compensation claims to pursuing dialogue with Government departments and individual companies on a range of matters. Ongoing education was an important activity particularly to reproduce technical information about asbestos in a form and in language which could be understood. The Auckland branch took up a wider issue which extended to non-work exposure to asbestos in schools and homes and the construction of homes on ground fills contaminated with dumped asbestos [49].

## 14. The Two Christchurch Earthquakes, The Pike River Mine Disaster 2010–2011

All three events have already been referred to and it is clear in hindsight they had a significant impact on subsequent departmental restructuring and asbestos legislation.

The urgent and widespread removal of demolished and damaged buildings, initially with little concern as to the nature of the demolition products whether concrete, brick, plaster, asbestos insulation or asbestos cement products, soon raised concerns among the public as convoys of trucks trundled past houses on their way to tip sites. This initial concern, which to some extent was accepted as an understandable response to the clean-up of damaged sites, was soon followed by a more ongoing asbestos issue, namely, the need to renovate and repair homes damaged by the two earthquakes. At this point, there was real householder concern as to the nature of the asbestos-containing ceilings and the care with which the work was carried out. The result was a major response by both the Ministry of Health and the Department of Labour.

The former initially relied on a document developed in 1997 [50]; this was later followed by the establishment of the Asbestos Technical Advisory Group (2014) to update the potential risk of asbestos exposure to those in non-work environments. One of the important outcomes was the request for an Inventory of New Zealand Imports and Exports of Asbestos-Containing Products [51]. The result was a Government decision to ban the importation of asbestos-containing products. 

At the same time the Department of Labour at Head Office established a number of internal groups to review issues ranging from upskilling the inspectorate on asbestos, developing new regulations on asbestos, to refining the register and training requirements of asbestos removal operators.

While on the ground in the Canterbury region, the Department recruited a senior manager to coordinate activities in the region. The key outcomes were the establishment of the Canterbury Rebuild Safety Charter [52], a request for a University-based literature review of health-related issues likely to arise in the construction industry during the rebuild [53] and an intense programme of workshops, breakfast meetings and seminars, to focus on asbestos-related health risks associated with on-going demolition, house renovation, and infrastructure repair. Later came tightening of building codes, requiring higher standards for civic and housing high-rise structures.

This accelerated period of Government activity also saw the Prime Minister request his Chief Science Advisor in association with The Royal Society of New Zealand to develop a report “Asbestos exposure in New Zealand: Review of the scientific evidence of non-occupational risks” [54], an important and landmark document.

While the two earthquakes had this huge impact on the issue of asbestos, between the two events a major explosion occurred in an underground coal mine on the West Coast at Pike River [55,56]. The consequent inquiry resulted in a decision by Government to review the health and safety function of the Department of Labour which resulted in the establishment of a new crown Entity, WorkSafe, to carry out the role of the Regulator in workplace health and safety.

## 15. The Health and Safety at Work Act 2015—The Asbestos Regulations 2016

By 2015 a new Health and Safety at Work Act was in place, this was followed by the Health and Safety at Work (Asbestos) Regulations. 2016, together with an Approved Code of Practice (ACOP) for The Management and Removal of Asbestos 2016, approved by the Minister of Workplace, Recreation and Safety under section 222 of the Health and Safety at Work Act 2015 [57].

Thus, in the relatively short period from 2010/2011 to 2015/2016, Government and in particular the Departments of Labour, the Ministry of Health, The Environmental Protection Agency and the Ministry for the Environment had achieved much in terms of banning the importation of products containing asbestos, coming to grips with the scourge of asbestos in the work and non-work areas and in introducing legislation and codes of practice to manage renovation, removal and demolition of environments containing asbestos.

## 16. Conclusions

The eighty-year saga was reaching its end. The events and influences have been discussed and have illustrated how leadership moved from one institution or organisation to another until finally those who developed the disease and died from asbestos exposure and their families, said “enough”, breaking their silence and going public. Without the, at times, passionate support from a small number of dedicated press reporters, their task would have been much harder. It took courage for both the victims, their families and the reporters to do what they did.

What of the medical profession? not known generally for speaking out on matters concerning workers’ health, their response was muted certainly initially. This undoubtedly reflected the lack of training in “work” as a factor in disease causation as well as the few cases of asbestos-related disease which occurred in the early years as latency hid the outcome. Even in the 1990s, radiologists were reporting on pleural plaques as asbestosis. 

Departments of Health and Labour also found it difficult to manage occupational diseases compared with injuries, so that too often occupational health fell to the bottom of the priority list. Sadly, departments of respiratory medicine were not free from this failing. The outcome was that diagnosis, notification, recording and analysis were inadequate and to ignore the problem became the easy option. However, over time, reports began to appear in local medical journals [58,59,60,61,62,63,64,65,66,67,68].

Ultimately, Government carried out its leading role and banned the importation of most products containing asbestos. It had responded to external pressures. There is a sense at the end that something other than an evidence base was necessary to achieve these changes, something deeper and more fundamental was involved. That is, respect for people who work, particularly for those who work in the hazardous trades, a compassion for those affected and a recognition that at the end, are we not our brother’s keepers?

Some suggestions arise from this paper which may be of an assistance to developing countries still facing the risks of asbestos use. Firstly, the importance of maintaining pressure on Governments to recognise and act to eliminate asbestos as a health risk. Secondly, to ensure there is public access to information about the health risks of asbestos. Thirdly, to encourage inter-country exchange of individuals, researchers and Government officials to share experience and solutions.
